# IgG4-related disease complicated by PLA2R-associated membranous nephropathy: A case report

**DOI:** 10.1515/biol-2022-0921

**Published:** 2024-07-25

**Authors:** Meichun Huang, Jun Liu, Xiuxiu Li

**Affiliations:** Renal Department, Tongde Hospital of Zhejiang Province, No. 234 Gucui Road, Hangzhou, 310012, China; Renal Department, Tongde Hospital of Zhejiang Province, Hangzhou, 310012, China

**Keywords:** IgG4-related disease, IgG4-related kidney disease, membranous nephropathy, phospholipase A2 receptor, *Tripterygium wilfordii*

## Abstract

IgG4-related tubulointerstitial nephritis (IgG4-related TIN) is the prevalent clinical manifestation of IgG4-related diseases (IgG4-RD). However, there are limited cases of IgG4-RD occurring with membranous nephropathy (MN) in the absence of phospholipase A2 receptor (PLA2R). There have been no indications of treatment using *Tripterygium wilfordii*. This study reported a rare case of IgG4-RD with PLA2R-associated MN without any of the distinct IgG4-related TIN. The patient was treated effectively with *T. wilfordii*. A 71-year-old patient was admitted to the medical facility after presenting with a 1 month history of edema and 8 months of albuminuria. The renal biopsy tissue examination confirmed the presence of MN (phase II) in the absence of pathological manifestations of IgG4-related TIN. Immunohistochemistry identified PLA2R++ (granular capillaries). The serum PLA2R antibody titer was 1:180 (1:20). The patient met the diagnosis with IgG4-RD. Over 8 years of follow-up, the patient was effectively treated with low-dose hormones and *T. wilfordii*, without any adverse effects. This MN is considered a unique form of IgG4-RD, regardless of whether PLA2R antibodies are present or not. Research suggests that *T. wilfordii* could be a promising option for elderly people with IgG4-related MN, as it has been found to have fewer adverse effects.

## 1 Introduction

IgG4-related diseases (IgG4-RD) are systemic disorders that can impact a wide range of organs. The diagnostic criteria for IgG4-RD were initially suggested in 2011, and then, an IgG4-RD classification system was introduced in 2019 [1,2]. IgG4-related tubulointerstitial nephritis (IgG4-related TIN) is the most common manifestation of kidney injury, characterized by dense lymphoplasmacytic infiltration (≥10 IgG4+ plasma cells/HP) and/or an IgG4+/IgG+ plasma cell ratio of ≥40%. There is limited evidence of IgG4-RD presenting with membranous nephropathy (MN), which is frequently accompanied by IgG4-TIN and is characterized without phospholipase A2 receptor (PLA2R) expression [1,2].

The understanding of IgG4-RD is constantly expanding, accompanied by investigations into their diagnosis and treatment. The link between these diseases and renal damage is complex. Multifactorial factors frequently contribute to the increasing prevalence of MN, which is recognized as an immune-related disease. In this report, a highly rare case of an IgG4-RD with PLA2R-associated MN was presented, which lacks the typical IgG4-related TIN. During the 8-year follow-up period, the patient experienced no recurrence or adverse effects after being treated with low-dose hormones in combination with *Tripterygium wilfordii*. The diagnosis of IgG4-RD in this patient was challenging and two diagnostic criteria were used. IgG4-RD was predicated upon the first criterion, whereas the diagnosis met the requirements of the second criterion. The diagnosis of IgG4-RD, the relationship between IgG4-RD and MN, and the role of PLA2R antibodies are also reviewed. More specifically among elderly patients, *T. wilfordii* is found to have therapeutic effects.

## 2 Case report

**Figure 1:**
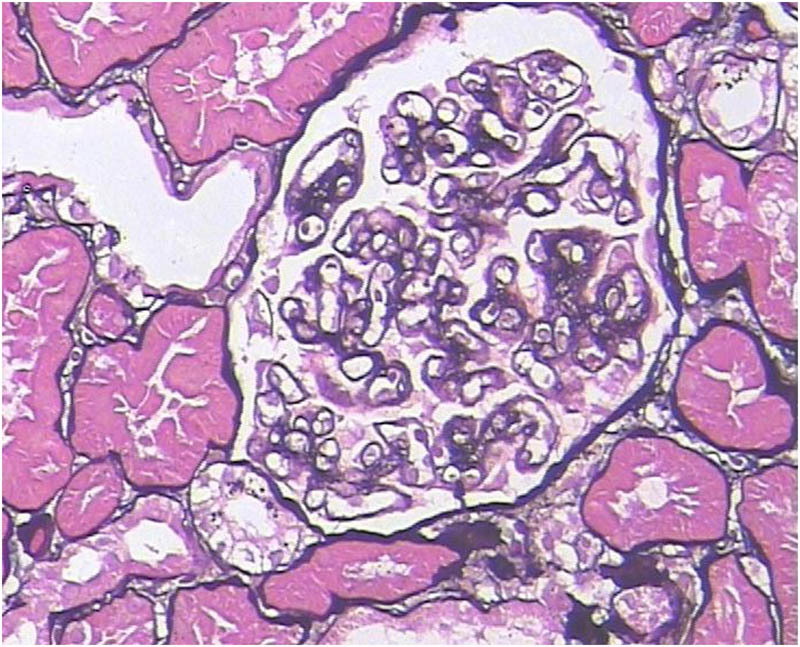
Histopathological examination of the kidney reveals MN via periodic acid Schiff-methenamine staining (400×).

**Figure 2:**
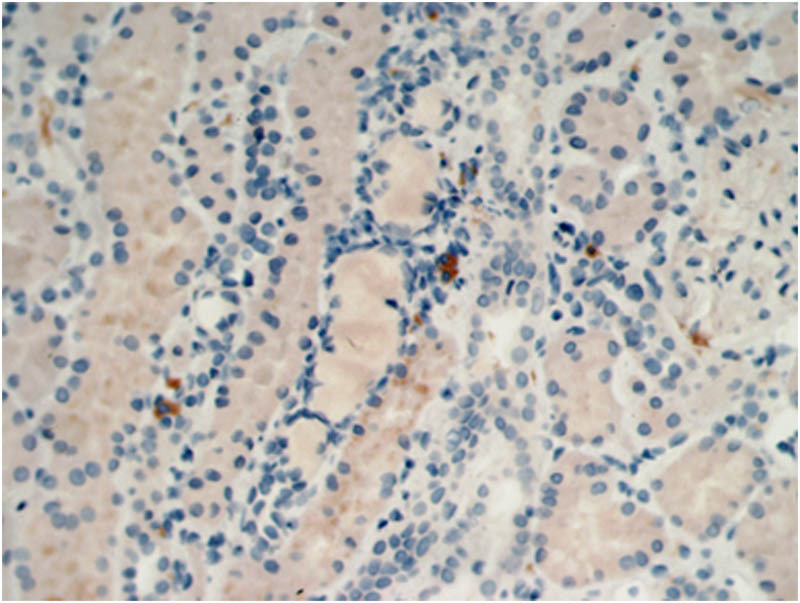
Immunohistochemical analysis of IgG4+ plasma cells < 10/high power field (Hp) (400×).

**Figure 3:**
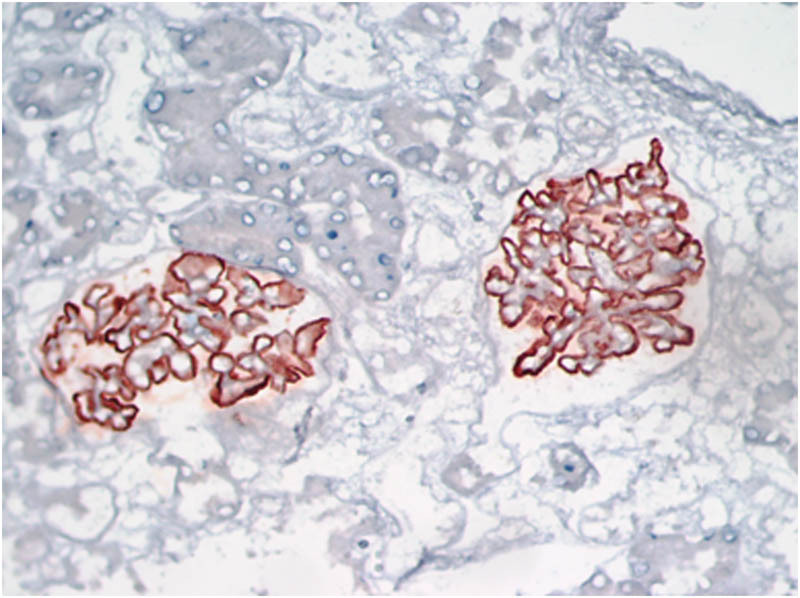
Immunohistochemical analysis of the kidney reveals PLA2R++ (400×).

A 71-year old male was admitted to the Nephrology Department on March 4, 2015, due to albuminuria persisting for 8 months and edema for 1 month, along with symptoms of fatigue, dry mouth, and polydipsia. Urine analysis showed red blood cells 1–3/HP, protein+++, and proteinuria 3500.00 mg/day. Serum analysis revealed albumin 24.4 g/L, creatinine 79 μmol/L (40–83 μmol/L), IgA: 1.74 g/L, IgM: 1.21 g/L, IgG: 20.68 g/L (7–16.00), IgG4: 9.95 g/L (0.03–2.01), IgG4/IgG: 48.11%, C3: 0.38 g/L (0.79–1.52), and C4: 0.04 g/L (0.16–0.38). The PLA2R antibody titer in serum was 1:180 (1:20). Changes in bilateral parotid echo were detected via ultrasound, whereas abdominal CT and enhanced MRI revealed that both the body and tail of the pancreas were enlarged. Blood eosinophils and IgE, thyroid function, tumor markers, antinuclear antibodies, and routine bone marrow tests were normal. The immunofluorescence for renal biopathology was examined on March 25, 2015. The main findings were as follows: 3 glomeruli, IgG+ (capillaries), C3++ (capillaries), IgM++ (capillaries), C1q++ (capillaries), IgA−, Fg−, and C4−. The light microscopy analysis revealed 29 glomeruli, of which 5 showed glomerulosclerosis and the remaining 29 displayed mild hyperplasia of the mesangial tissue, basement membrane thickening, double tracks, nail processes, renal tubule atrophy, rare interstitial fibrous tissue hyperplasia, and inflammatory cell infiltration (≤5%) (Figure 1). Diffuse fusion of foot processes, basement membrane hypertrophy, mesangial matrix enlargement, and accumulation of electron-dense matter beneath the epithelium, within the basement membrane, and in the mesangial region were all observed via electron microscopy. Immunohistochemistry showed HBsAg (−), HBcAg (−), K (−), L (−), focal distribution of CD138-positive plasma cells in the renal interstitium with an average of 18/Hp, IgG4-positive plasma cells < 10/Hp (Figure 2), and PLA2R++ (capillaries, granular) (Figure 3). The pathological symptoms of IgG4-related TIN were not indicated by the diagnosis of MN (phase II).

**Figure 4:**
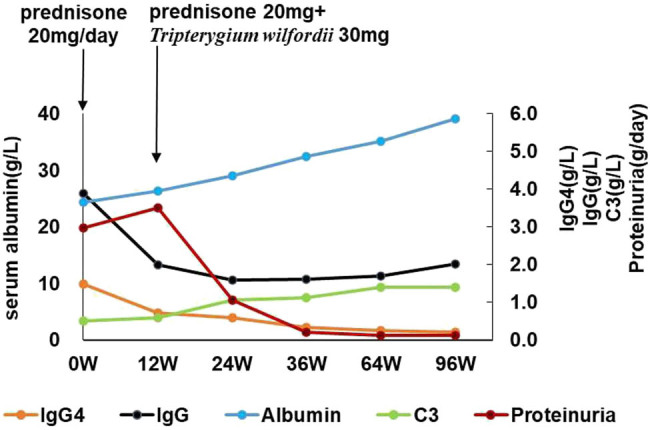
Disease progression is monitored by quantifying serum proteinuria, serum albumin, serum IgG4 level, serum IgG level, and serum C3 level. *T. wilfordii* were administered after the initiation of prednisolone therapy. W = week.


Based on the patient’s clinical symptoms (dry mouth and excessive drinking, bilateral abnormalities in the parotid glands, significant enlargement of the pancreas, increased serum IgG4 level, and hypocomplementemia), the patient met all inclusion and exclusion criteria and obtained a total score of 26 points (≥20 points) on the IgG4-RD classification standard. Thus, a clinical diagnosis of IgG4-RD was determined by the classification criteria of the European League Against Rheumatology and the American College of Rheumatology. This led to the diagnosis of IgG4-RD in combination with MN for the patient. The patient received prednisone (20 mg/day) treatment on March 5, 2015. IgG4 levels had decreased to 4.77 g/L, while serum IgG and parotid gland levels had returned to normal after 3 months. However, the nephrotic syndrome remained unresolved. After this, the patient was prescribed a daily dose of prednisone (20 mg) along with *T. wilfordii* (30 mg). After 2 months, the urine protein test was negative, which led to a gradual decrease in dosage. Finally, a daily dose of prednisone (5 mg) and *T. wilfordii* (10 mg) was given and continued, until it was stopped 1 year ago (Figure 4). The serum anti-PlA2R became negative. All clinical indicators remained within normal ranges in the 8-year follow-up period.

**Informed consent:** Informed consent has been obtained from all individuals included in this study.
**Ethical approval:** The research related to human use has been complied with all the relevant national regulations, institutional policies and in accordance with the tenets of the Helsinki Declaration, and has been approved by the authors’ institutional review board or equivalent committee.

## 3 Discussion

Based on the diagnostic criteria from 2011, the patient was identified as having suspected IgG4-RD during his 2015 visit. However, the diagnosis of IgG4-RD remained possible following 8 years of follow-up observation and the use of the 2019 revised classification criteria. However, the patient also developed nephrotic syndrome, which was characterized by the pathological presentation of MN but lacked evidence of IgG4-TIN. There was a positive finding of PLA2R in both the serum and renal tissue, while electron microscopy revealed the presence of electron-dense deposition commonly observed in atypical MN.

Currently, there is a gradual increase in the incidence rate of MN in glomerular diseases. Despite secondary MN is frequently observed as a pathological symptom, its underlying cause is often unclear. Based on the results, the serum anti-PLA2R antibody level showed remarkable sensitivity (68%) and specificity (97%) in differentiating primary and secondary MN. The PLA2R antibody level is a characteristic indicator of primary MN with low positivity in secondary MN [3–5]. Recently, there have been a few reports of IgG4-RD associated with MN, which may be IgG4-related MN. Most of these cases are negative for PLA2R antibodies, and IgG4-TIN often occurs simultaneously or progressively. Peking University Hospital reported 42 patients with IgG4-related kidney disease (IgG4-RKD), with an average age of 58.5 ± 8.7 years (male to female ratio = 5:1). Among them, 66.7% showed acute kidney injury. IgG4-related TIN was identified in 40 patients, of which 19 (47.5%) had concurrent glomerular pathologies. The patients with MN received prednisone (0.5–1.0 mg/kg/day) and or combined with Cyclophosphamide or Cyclosporine. In another study, IgG4-related TIN and MN were observed in 27.4% of the patients. IgG4-related MN can have rare manifestations associated with tumors, monoclonal globulinemia, systemic lupus erythematosus, and secondary infection with the EB virus. The etiology of IgG4-related MN remains largely unknown [6,7].

Most patients with IgG4-related MN are negative for anti-PLA2R, with rare reports of anti-PLA2R-positivity. A patient who had previously experienced pancreatitis associated with IgG4 was admitted to the hospital with nephrotic syndrome; upon pathological examination, positive serum anti-PLA2R antibody levels indicated MN without interstitial nephritis. The patient was treated with hormones combined with Cyclophosphamide. A case of pancreatic and liver damage with typical IgG4-RD was reported. MN developed nephrotic syndrome during the active phase of the disease 5 months later. Serum and renal tissue were both positive for anti-PLA2R via immunofluorescent analysis. MN experienced relief after Rituximab treatment. A 55-year-old woman was reported to have been diagnosed with MN in combination with IgG4-RD in the absence of interstitial nephritis in 2022. The renal tissue was positive for anti-PLA2R. Despite a reduction in IgG4 levels after hormone therapy, the nephrotic syndrome persisted and was effectively treated with Obinutuzumab [8,9].

These patients were diagnosed with IgG4-RD concurrent with PLA2R-related MN, without interstitial nephritis. It is evident from these patients that PLA2R-associated MN may occur either after IgG4-RD or at the same time, without interstitial nephritis generally. The efficacy of hormone therapy alone is not satisfactory. However, after immunosuppression or combined treatment with biological agents, the prognosis may be good.

Currently, it is challenging to determine if the associated MN indicates idiopathic PLA2R-related MN or IgG4-related MN.

What is the mechanism between PLA2R-related MN and IgG4-RD? There is a genetic predisposition to the primary MN, with a genetic interaction of a locus in or near an enhancer region of the PLA2R1 gene itself and the larger class II major histocompatibility complex locus. PLA2R was expressed by normal human podocytes. In disease, PLA2R binds to their *in situ* or circulating immune complexes on the podocyte, leading to the accumulation of immune complexes, which cause gradual accumulation of the deposits beneath the podocyte (in a subepithelial position). It is not clear why this occurs in discrete areas, leading to more complexes in some locations but not others. The clinical utility of the identification of PLA2R is not only a specific way to diagnose PLA2R-related MN [4,5] but also the prediction of the subsequent clinical course. The formation of immune complexes with IgG4 seems likely to be described in rheumatoid arthritis, IgG4-RD, and MN. The pathogenesis of IgG4-RD assumes the increased activity of T regulatory cells with overexpression of IL-10, production of transforming growth factor-β, and upregulation of Th2 response, in which interleukins 4, 5, and 13 play predominant roles. These cytokines translate into specific pathophysiological phenomena such as the activation of B cells and plasma cells to IgG4 production. So, B- and T-cells are vital to disease pathogenesis [1,5]. The mechanism is unclear in which PLA2R-immune complexes are activated in the pathological process of IgG4-related MN.

We present a rare case of PLA2R-associated MN concomitant with IgG4-RD. However, electron-dense subepithelial, endosubcutaneous, and mesangial deposits suggested a secondary etiology for MN.

During the 8 years follow-up, the patient did not develop any membranous kidney-related tumors, poisoning, thyroid diseases, or infections. With the remission of IgG4-RD disease, nephrotic syndrome did not recur and the PLA2R titer did not increase again. Previous studies showed the presence of deposits in the subendothelial or mesangial regions, or both, in people with IgG4-related MN. Therefore, although the present case was PLA2R-positive, it is believed that the MN may be a secondary IgG4-RD. In IgG4-RD, serum IgG4 levels decline after B cell depletion but do not normalize, probably because of the long-lived plasma cells that continue to produce it, which results in persistently elevated immunoglobulin. Therefore, the patient’s treatment with only hormone therapy is ineffective. However, a significant improvement resulting in complete remission without recurrence, was noted after *T. wilfordii* treatment.

Polyglycosides from *T. wilfordii* are extracted from the root core, which has been widely used for the treatment of IgA nephropathy, MN, and diabetic nephropathy, as well as rheumatoid arthritis, systemic lupus erythematosus, and other immune diseases [10–12]. The therapeutic activity of *T. wilfordii* triptolide is mediated by the PI3K/AKT/mTOR pathway to alleviate membranous nephropathy [13]. Molecular biological research on the treatment of MN with *Tripterygium* glycosides, according to the principles of traditional Chinese medicine, the effects of treatment with *Tripterygium* glycosides mainly involves the AGE-RAGE signaling pathway, lipids and atherosclerosis, the IL-17 signaling pathway, fluid shear stress, atherosclerosis, and the NF-κB signaling pathway to regulate the release of inflammatory factors and T cell proliferation [13–15]. *Tipterygium* glycosides can also suppress the Toll-like receptor 4 pathway, while triptolide can inhibit both cell proliferation and inflammatory cytokine expression in IL-6/sIL-6R-stimulated fibroblast-like synoviocytes by suppressing JAK2/STAT3 signaling and reduce the levels of TNF-α, IL-8, CXCL2, and VEGF [12,15,16]. After treatment of patients with chronic nephritis with *T. wilfordii* polyglycoside tablets, both the CD4^+^ and CD4^+^/CD8^+^ ratios increased, while that of CD8^+^ decreased [17]. *Triptolide* reduced podocyte injury via microRNA-155-5p/brain-derived neurotrophic factor [11]. *T. wilfordii* was effective in preventing T cell proliferation and regulated the balance of Tfr/Tfh through inhibiting proliferating B cells [18]. *Tripterygium* glycosides can improve abnormal lipid deposition in nephrotic syndrome, and decrease renal oxidative damage and apoptosis [19]. So, *T. wilfordii* has a potential multi-component, multi-target, and multi-pathway molecular mechanism of action for the treatment of MN [20]. According to a study on the efficacy and safety of *T. wilfordii* multiglycosides in idiopathic membranous nephropathy, the reported incidence of reproductive toxicity and hepatotoxicity among users of *T. wilfordii* multiglycoside tablets (1–1.5 mg/kg body weight/day, orally, divided to three doses) was less [21,22]. Even the adverse effects were mild and reversible. The use of excessive doses is the main cause of adverse effects. We selected a 30 mg/day low-dose treatment, which has no adverse effects and has good clinical efficacy.

## 4 Conclusion

This study concluded that MN is a unique form of IgG4-RD, irrespective of whether PLA2R antibodies are present or not. Studies suggest that *T. wilfordii* could offer potential benefits for elderly people with IgG4-MN, due to its therapeutic impact and reduced risk of side effects.
